# Appointments

**Published:** 1855-10

**Authors:** 


*Appointments.*—Dr. Sanford B. Hunt (editor of this Journal) has been appointed one of the Attending Physicians at the Buffalo Hospital of the Sisters of Charity, for the winter term.

Dr. John Boardman (Demonstrator of Anatomy in the University of Buffalo) has been appointed one of the Attending Surgeons in the same institution, for the same term.

